# MC TRIM Algorithm in Mandibula Phantom in Helium Therapy

**DOI:** 10.3390/healthcare11182523

**Published:** 2023-09-12

**Authors:** Fatih Ekinci, Koray Acici, Tunc Asuroglu, Busra Emek Soylu

**Affiliations:** 1 Institute of Nuclear Sciences, Ankara University, Ankara 06830, Turkey; fatihekinci@ankara.edu.tr; 2 Artificial Intelligence and Data Engineering, Ankara University, Ankara 06830, Turkey; 3 Faculty of Medicine and Health Technology, Tampere University, 33720 Tampere, Finland; 4 Computer Engineering Department, Ankara University, Ankara 06830, Turkey

**Keywords:** helium ion therapy, Monte Carlo, TRIM algorithm, mandible plate phantom, polymeric biomaterials

## Abstract

Helium ion beam therapy, one of the particle therapies developed and studied in the 1950s for cancer treatment, resulted in clinical trials starting at Lawrence Berkeley National Laboratory in 1975. While proton and carbon ion therapies have been implemented in research institutions and hospitals globally after the end of the trials, progress in comprehending the physical, biological, and clinical findings of helium ion beam therapy has been limited, particularly due to its limited accessibility. Ongoing efforts aim to establish programs that evaluate the use of helium ion beams for clinical and research purposes, especially in the treatment of sensitive clinical cases. Additionally, helium ions have superior physical properties to proton beams, such as lower lateral scattering and larger LET. Moreover, they exhibit similar physical characteristics to carbon, oxygen, and neon ions, which are all used in heavy ion therapy. However, they demonstrate a sharper lateral penumbra with a lower radiobiological absence of certainties and lack the degradation of variations in dose distributions caused by excessive fragmenting of heavier-ion beams, especially at greater depths of penetration. In this context, the status and the prospective advancements of helium ion therapy are examined by investigating ionization, recoil, and lateral scattering values using MC TRIM algorithms in mandible plate phantoms designed from both tissue and previously studied biomaterials, providing an overview for dental cancer treatment. An average difference of 1.9% in the Bragg peak positions and 0.211 mm in lateral scattering was observed in both phantoms. Therefore, it is suggested that the ^4^He ion beam can be used in the treatment of mandibular tumors, and experimental research is recommended using the proposed biomaterial mandible plate phantom.

## 1. Introduction

Heavy ion therapy is playing an increasingly important role in cancer treatment, along with pioneering treatments such as protons and carbon ions [1,2,3]. Comprehensive clinical trials at Lawrence Berkeley National Laboratory have investigated the physical properties of different particle types, including protons, helium ions, neon ions, and argon ions [4]. Carbon ion beams, with their higher linear energy transfer (LET) values, have been explored for carbon ion therapy in Japanese and German facilities to enhance dose conformity and biological effectiveness [5]. However, protons and carbon ions are not considered “perfect” particles, and experimental and simulation studies are ongoing to determine the most suitable ion type for a specific indication [2,6,7].

In this context, there is a growing interest in helium ion beams, and various studies are exploring their unique biophysical properties that lie between the two main clinical modalities [5,6]. Despite their favorable physical and biophysical characteristics, helium ions are not yet used clinically worldwide, and are limited to experimental purposes only [5,8]. Helium ions are believed to provide increased linear energy transfer (LET) and targeting with clinically beneficial distributions compared to protons [6,9]. Moreover, taking into account the possibility of a streamlined facility layout with a reduced biological effectiveness degradation tail/variability compared to carbon ions, the expected clinical benefits will offer numerous untapped medical and financial advantages [10]. Additionally, ^4^He ion beams exhibit a diminished lateral scattering and penumbra comparable to carbon ion beams, accompanied by a substantial decrease in the fragmentation tail at higher clinical energies [6,9,11]. With these attributes, ^4^He ion therapy has the potential to enhance clinical effectiveness across diverse treatment areas, such as meningiomas and pediatric conditions [12,13].

Monte Carlo (MC) simulation methods are extensively utilized in heavy-ion therapy to model and predict mixed radiation fields [5]. The precise characterization of combined radiation fields for different types of particles is essential for creating a simulation framework and forecasting the physical and effective dose in intricate scenarios, such as patient geometries [14]. In recent years, significant progress has been made in the field of MC simulation methods with the development of advanced codes such as FLUKA [15,16,17], GEANT4 [18,19], PHITS [20,21], and TRIM [6]. These codes have played a role in improving the modeling of ^4^He ion beam radiation and have been revised with cross-sectional measurements to gain a deeper understanding of helium ion fragmentation behavior. The intricacy of the head and neck region poses treatment challenges due to the proximity of tumors to adjacent normal tissues [22,23]. Heavy ion therapy can impact various neoplastic conditions in the lower facial region, particularly odontogenic tumors [24]. The jawbones, mandible, and maxilla can encompass not only primary tumors but also secondary lesions such as oral cancers and metastatic lesions [25]. Managing locally advanced cancers in the oral cavity and paranasal sinuses presents notable challenges in treatment [26]. One of the side-effects of radiotherapy, especially in dental cancer treatment, is the occurrence of radiation-induced osteomyelitis or osteoradionecrosis in the jawbone [27]. Oral complications associated with radiation therapy include oral mucositis, taste disturbances, infectious diseases, and xerostomia due to salivary gland dysfunction [28]. The incidence of these complications increases with higher radiation doses. Methods like intensity-modulated radiation therapy, which optimizes dose distribution, have improved treatment outcomes by selectively preserving organs at risk and reducing clinical toxicity [29,30,31]. In this regard, research conducted on phantoms before treatment has gained significant importance. Phantoms that accurately simulate the jawbone and use tissue-equivalent biomaterials have recently garnered attention [32].

In this study, Bragg curves of therapeutic-energy ^4^He ion beams were calculated for simulated mandibular plate phantoms using real tissue and biomaterials. The results were compared with other calculations in the literature and showed acceptable deviations within the tolerance range. Finally, Bragg peak positions as well as lateral range/scattering and rebound curves were obtained for both mandible plate phantoms. Using the results obtained, an attempt was made to determine the appropriate ^4^He energy corresponding to the location of the tumor. In addition to the lateral scattering and range values, the beam scattering in the mandibular tissue layers was also determined. At this point, the results were compared with those obtained using a mandibular biomaterial phantom. Thus, in addition to investigating the use of ^4^He ions in dental cancer, a dental phantom obtained from biomaterials was proposed for experimental research. Thus, in experimental studies, an attempt was made to obtain realistic data with the help of the phantom created in biomaterial tissues closest to the real tissue.

## 2. Materials and Methods

The identification and outlining of critical organs with designated dose limitations were conducted, including the cheek, masseter muscle, parotid gland, oral mucosa, gingiva, mandibular cortical and spongy bone, saliva, air, and SMAS. The assessment of density and thickness for each contour relied on established studies found in the literature [33] (Rosenthal, 2008). In this particular study, the tissue thickness in the region encompassing tooth molars and premolars was assessed, leading to the identification of two distinct optimal dosages for these regions. The MC-based TRIM (TRansport of Ions in Matter) simulation program was utilized to compute the Linear Energy Transfer (LET) of ^4^He within the phantom layer material. TRIM leverages quantum mechanical methods to accurately estimate the ion stopping power and range within a target, encompassing all relevant kinetic events associated with ionization and energy loss phenomena [34]. The layer type to be chosen is either created by using the periodic table or by using the mass–composition ratios of the elements. The layer thickness is formed with a thickness length from A to m. It allows for choosing the type of ion to be selected, the number of ions in the beam, and the energy of the beam. TRIM simulation offers a wide choice of calculation methods. It helps to choose methods in which many parameters are calculated. It can give the calculation results either in text files or in image format.

**Figure 1:**
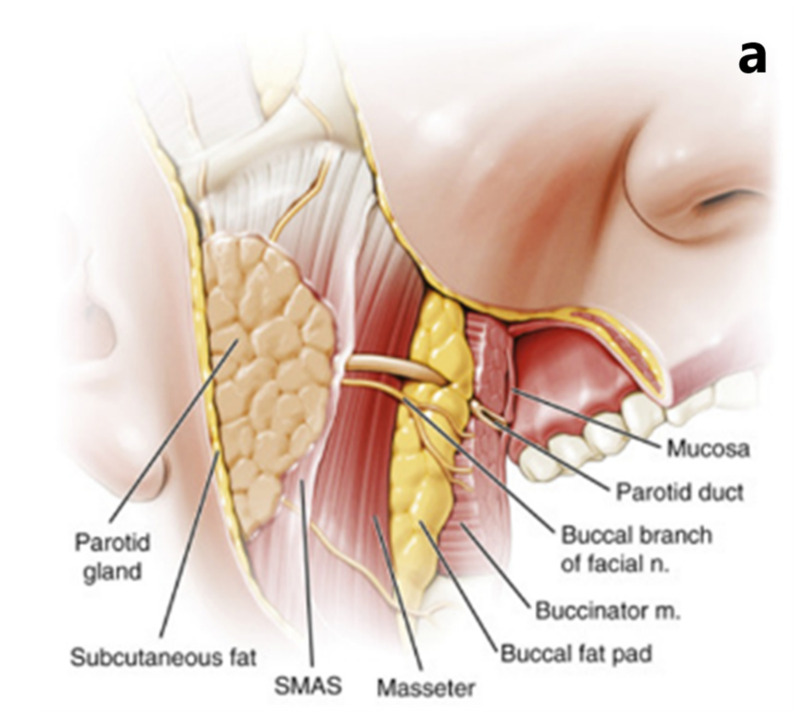

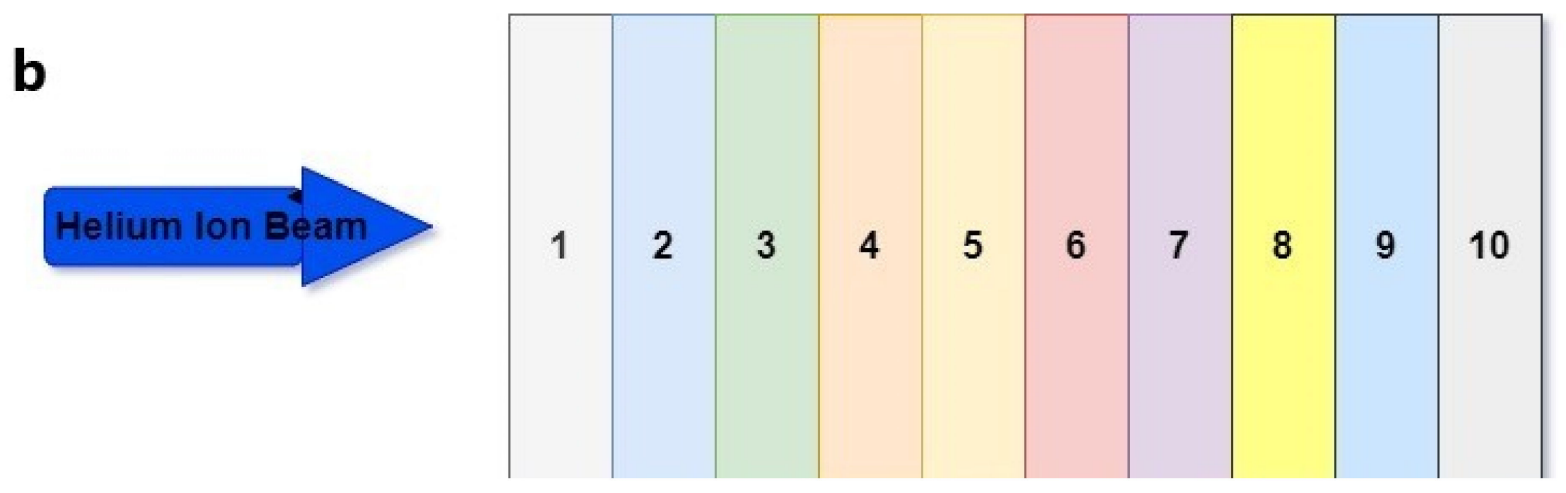
Transverse cross-section of the focused oral region mandible plate phantom [14] (**a**) and the visualization of the mandible plate phantom layers (1–10) (**b**) are provided. The layers are described in detail in Table 1.

In the calculations, a ^4^He beam (consisting of 10^5^ ions) was directed at the target with acceptable statistical deviations. The calculations were performed by considering the layer conditions created from biological and biomaterial structures based on the phantom structure shown in Figure 1. Two mandible phantoms were created, one from real tissues and the other from biomaterials, considering the physiology of the biological layers, including their atomic density, thicknesses, and mass, using plate cross-sections. These layers were the skin, parotid gland, SMAS, masseter muscle, buccal fat, mucosa, saliva, gum, cortical bone, and cancellous bone, respectively. All coatings constituting the jawbone were assigned realistic thicknesses using properties from the TRIM database. The chemical composition, atomic densities, and mass densities of all phantom tissue layers comprising the jawbone were extracted from the MC TRIM compounds database and are presented in Table 1. The selected biomaterials were chosen from the most suitable polymeric biomaterials to be used instead of soft tissue and hard tissue [6,9,22]. Polymeric biomaterials such as polytetrafluoroethylene (ICRU-227) (Teflon) [22] were chosen instead of hard tissues, and biopolymeric materials such as polymethyl methacrylate (PMMA), Paralene_N, and polyethylene were selected instead of soft tissues [6,9,22].

The primary innovation in this study lies in the parameters associated with recoil and lateral scattering, as defined by Kinchin–Pease (K-P) theory. This theory is employed to express these parameters, which are then utilized to compute the displacements that occur during the interaction between an ^4^He ion beam and a target material atom. [35]. The process initiates with the collision between a Primary Knock-on Atom (PKA) and a stationary atom, leading to the generation of two atoms in motion. The PKA retains a residual energy of *T − ε*, while the impacted atom acquires an energy of *ε − E_d_*, as indicated in previous research [36]:

*v*(*T*) = *v*(*T* − *ε*) + *v*(*ε* − *E_d_*) (1)

In the provided scenario, *E_d_* denotes the energy consumed in the reaction. By neglecting the impact of *E_d_* in comparison to ε, particularly when *ε* ≫ *E_d_*, it is assumed that when an atom with an initial energy *T* undergoes a collision and emerges with energy *T’*, while generating a new recoil with energy *ε*, no energy is transferred to the lattice. In this case, the relationship *T = T’ + ε* holds, resulting in the transformation of Equation (2) [36].

*v*(*T*) = *v*(*T* − *ε*) + *v*(*ε*) (2)

The limitation of Equation (2) in determining *v(T)* arises from the unknown energy transfer *ε*. As PKA and lattice atoms are identical, *ε* can vary between *0* and *T*. However, if the probability of energy transfer within the (*ε*, *d_ε_*) range during a collision is known, multiplying Equation (2) by this probability and integrating over all permissible values of ε can provide the average number of displacements. In accordance with the hard sphere model, the energy transfer cross-section is defined by Equation (3) [36]:

(3) $$\sigma \left(T,\varepsilon \right)=\frac{\sigma \left(T\right)}{\gamma T}=\frac{\sigma \left(T\right)}{T}$$

for atoms of the same type, and the probability of PKA with energy *T* transferring energy within the range (*ε*, *d_ε_*) to the impacted atom can be expressed as follows (Equation (4)) [36]:

(4) $$\frac{\sigma \left(T,\varepsilon \;\right)d_{\varepsilon }}{\sigma \left(T\right)}=\frac{d_{\varepsilon }}{T}$$

for *γ* = 1 (like atoms). By multiplying the right-hand side of Equation (2) by *d_ε_/T* and integrating from 0 to T, we obtain Equation (5) [36]:

(5) $$v\left(T\right)=\int_{0}^{T}\left[v(T-\varepsilon )+v(\varepsilon )\right]d\varepsilon =\frac{1}{T}\left[\int_{0}^{T}v(T-\varepsilon )d_{\varepsilon }+\int_{0}^{T}v(\varepsilon )d_{\varepsilon }\right]$$

By performing a change of variables from ε to *ε′ = T − ε* in the first integral of Equation (5), we obtain [36]

(6) $$v\left(T\right)=\left[\frac{1}{T}\int_{0}^{T}v(T-\varepsilon ’)d_{\varepsilon ’}+\frac{1}{T}\int_{0}^{T}v(\varepsilon )d_{\varepsilon }\right]$$

which essentially represents the sum of two identical integrals [36]. Hence,

(7) $$v\left(T\right)=\frac{2}{T}\int_{0}^{T}v\left(\varepsilon \right)d_{\varepsilon }$$

Before we proceed to solve Equation (7), it is important to analyze the characteristics of *ν*(*ε*) in the vicinity of the displacement threshold, *E_d_*. It is evident that when the kinetic energy *T* is less than *E_d_*, there will be no displacements [36]:

(8) $$v\left(T\right)=0\;for\;0<T<E_{d}$$

when *T* is equal to or greater than *E_d_* but less than 2*E_d_*, two potential outcomes can occur. The first possibility is that the impacted atom becomes displaced from its lattice site, while PKA, with a reduced energy below *E_d_*, takes its place. Alternatively, if the initial PKA fails to transfer *E_d_*, the impacted atom remains stationary, leading to no displacement. In both scenarios, only one displacement can occur when the PKA’s energy falls within the range of *E_d_* to 2*E_d_* [36].

(9) $$v\left(T\right)=1\;for\;E_{d}\leq T<E_{d}$$

By utilizing Equations (8) and (9), we can partition the integral in Equation (7) into three ranges: from 0 to *E_d_*, *E_d_* to 2*E_d_*, and 2*E_d_* to *T*. We can then assess and calculate the following expression [36]:

(10) $$v\left(T\right)=\frac{2}{T}\left[\int_{0}^{E_{d}}0d\varepsilon +\int_{E_{d}}^{2E_{d}}1d\varepsilon +\int_{E_{d}}^{T}v(\varepsilon )d\varepsilon \right]=\frac{{2E}_{d}}{T}+\frac{2}{T}\int_{{2E}_{d}}^{T}v\left(\varepsilon \right)d\varepsilon$$

To solve Equation (10), we can multiply it by *T* and differentiate both sides with respect to *T*, leading to [36]

(11) $$T\frac{dv}{dT}=v\;$$

with the corresponding solution being [36]

(12) v=CT

Extracting Equation (12) into Equation (10) gives [36]

(13) $$v=\frac{1}{E_{d}}$$

and hence [36]:

(14) $$v\left(T\right)=\frac{1}{{2E}_{d}}\;for\;{2E}_{d}\leq T<E_{c}$$

The maximum number of displacements is determined by *E_c_*, which represents the cutoff energy for electron stopping. If the energy of PKA exceeds *E_c_*, no additional displacements occur until the PKA’s energy decreases to *E_c_* as a result of electron energy losses. For energies below *E_c_*, electronic stopping is disregarded, and only atomic collisions are taken into account. When a PKA with *T > E_c_* is generated, the number of displacements is given by *v(T) = E_c_/*2*E_d_*. Thus, the complete outcome based on the K-P theory is as follows [36]:

(15) $$v\left(T\right)=\left\{\begin{array}{c} 0\\ 1\\ \frac{T}{{2E}_{d}}\\ \frac{E_{c}}{{2E}_{d}}\; \end{array}\right.\begin{array}{c} T<E_{d}\\ E_{d}\leq T<{2E}_{d}\\ {2E}_{d}\leq T<E_{c}\\ T\geq E_{c} \end{array}$$

It is crucial to highlight that by ignoring the threshold displacement energy, *E_c_, T/*2*E_d_* represents a genuine average, considering that the number of displacements can range from 0 (no energy transfers above *E_d_*) to *T/E_d_ −* 1 (each collision transferring just enough energy). For large values of *T*, *T/E_d_ >>* 1 [36]. Hence, the maximum value of *v(T)* is *T/E_d_*. The comprehensive displacement function, as defined by Equation (15) [36], encompasses these considerations.

The Norgett–Robinson–Torrens (NRT) model, proposed by Norgett, Robinson, and Torrens [37], is a widely used formula for estimating the number of PKA produced through displacement events. The NRT model provides a means to calculate the quantity of displaced atoms, according to the following equation:

(16) $$N_{v}\left\{\begin{array}{cc} 0 & E_{v}<E_{d}\\ 1 & E_{d}<E_{v}<2.5E_{d}\\ \frac{0.8E_{v}}{2E_{d}} & E_{v}<2.5E_{d} \end{array}\right\}$$

In Equation (16), *E_v_* represents the energy associated with damage, while *E_d_* refers to the threshold displacement energy [35]. Hence, parameters related to recoil and collision events are determined. Moreover, *E_d_* represents the energy dissipated by each recoiling target atom as it moves away from its lattice position and recoils within the target material [36]. The typical range for *E_d_* is typically around 1–3 eV, although specific values are unknown for most compounds. It is presumed that this energy is absorbed by phonons [36].

Another aspect examined in this study is lateral scattering. In this regard, *x_i_* represents the projection range of ion *i* along the *x*-axis, Σ*_i_x_i_* represents the sum of ion projection ranges, Σ*_i_x_i_/N* denotes the average projection range of *N* ions, and *<x>* signifies the mean projection range of all ions [34]. Similarly, the transverse coordinate y is treated in a similar manner, but with distances measured in the *XY* plane [34]. Therefore, lateral scattering can be defined as follows:

σ = [(Σ*_i_x_i_*^2^)/N − R_p_^2^]^1/2^ = <(Δ*x_i_*)^2^>^1/2^ (17)

In the case of a beam of projectile ions, assuming cylindrical symmetry in the energy deposition, it is expected that the average lateral displacement is zero (i.e., *R_y_ =* 0) [34]. Furthermore, the calculated ranges in the *Y* and *Z* directions are averaged to improve the accuracy of the calculations [34]. Lateral scattering can be mathematically expressed as follows:

σ*_y_* = [Σ*_i_*((|*y_i_*|+|*z_i_*|)/2)^2^/N]^1/2^ (18)

where *x* represents the lateral scattering, while *y_i_* and *z_i_* represent the projections of the *i*^th^ ion on the *Y* and *Z* axes, respectively. In summary, this study focuses on the parameters and effects of recoil and collision events in ion–beam interactions. The K-P theory is utilized for determining the atom displacements, considering energy transfer probabilities and cross-sections. The NRT model is employed for calculating the quantity of displaced atoms. This study also explores lateral scattering, considering the projection ranges and average scattering distances of ions.

## 3. Results

### 3.1. Bragg Curves

**Figure 2:**
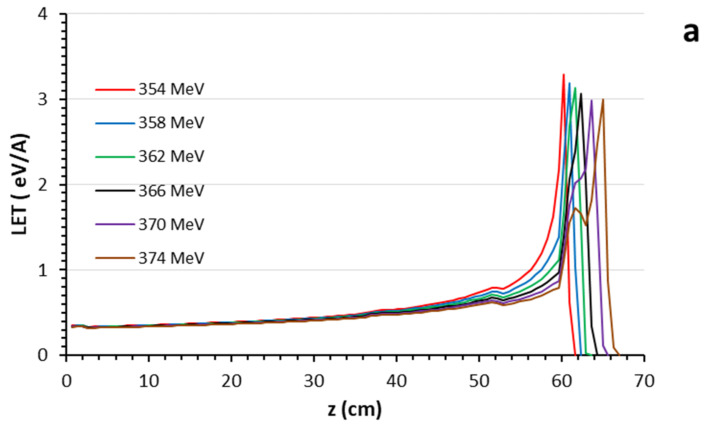

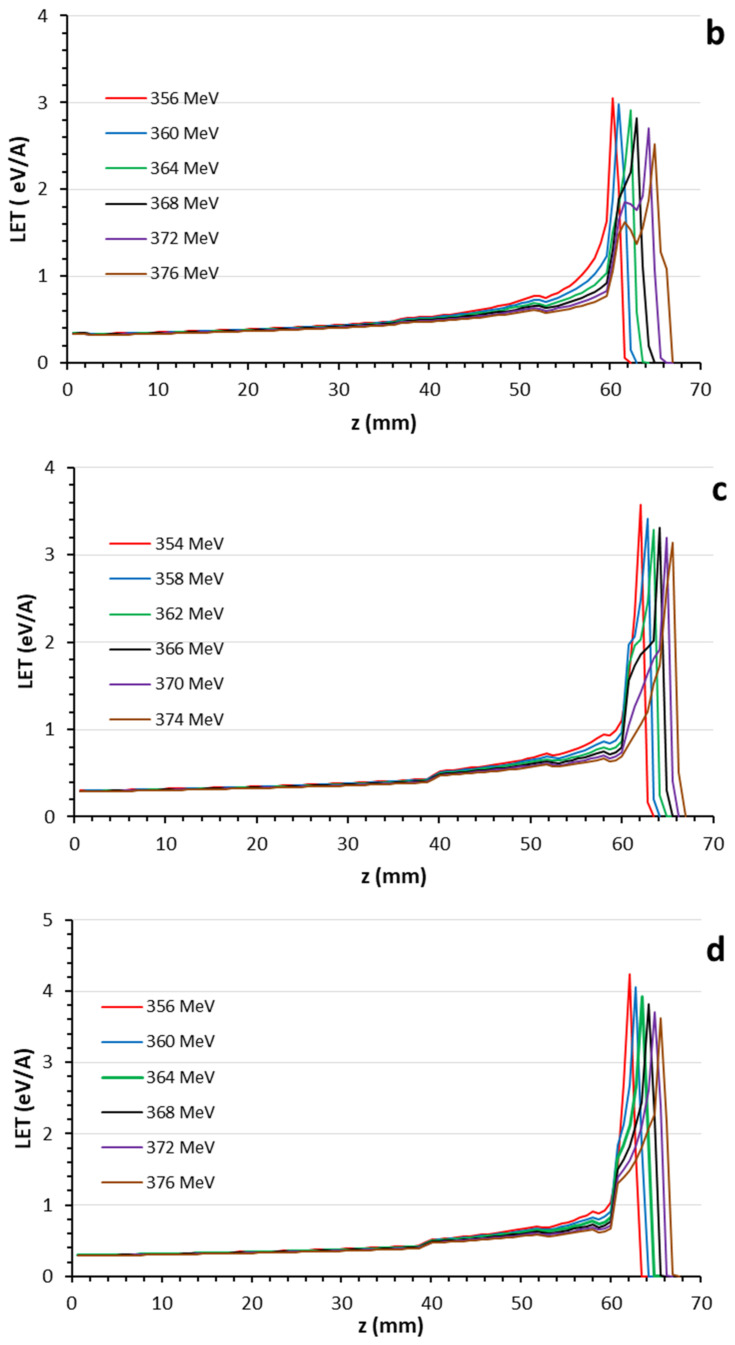
Bragg curves generated by 354–376 MeV energy ^4^He ion beams in mandible plate phantoms created using real tissues (**a**,**b**) and biomaterials (**c**,**d**).

The Bragg curves generated by the ^4^He ion beam were obtained using a mandible plate phantom without the use of layers created from real tissues and biomaterials. The variations in all layers of the mandible plate phantom were examined by incrementing the energies of 354–376 MeV ^4^He ion beams by 2 MeV. These Bragg peaks are presented in Figure 2. Figure 2a,b show the Bragg peaks observed in the mandible phantom created using real tissues, while Figure 2c,d show the Bragg peaks obtained in the mandible phantom created using biomaterials.

**Figure 3:**
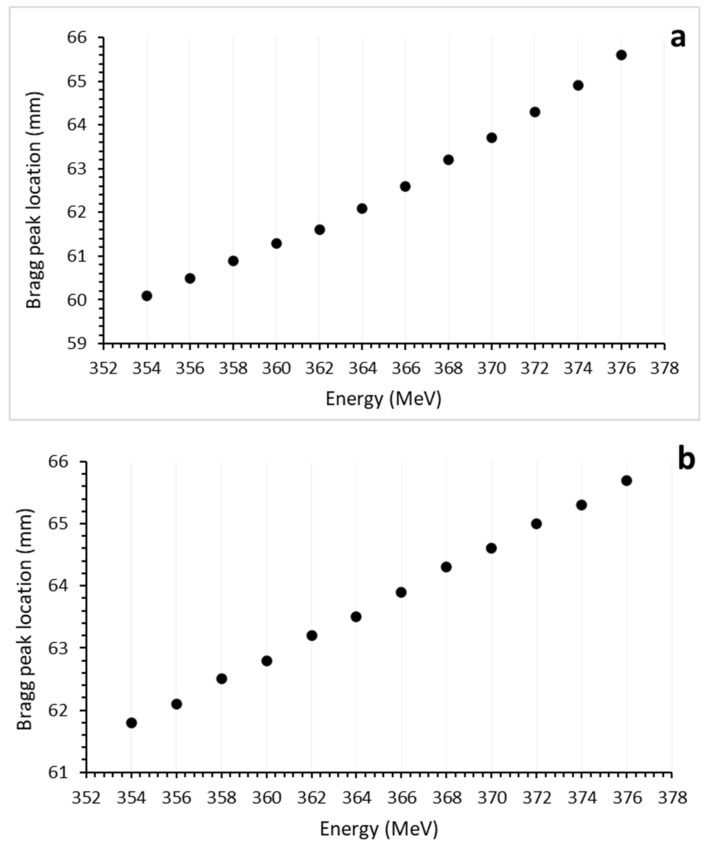
Bragg peak positions of ^4^He ion beams in mandible plate phantoms created from tissue (**a**) and biomaterial (**b**) layers.

When comparing the Bragg peak positions between the phantom created using real tissues and the one created using biomaterials, an average difference of 1.8% was found. This observed difference was found to be below the accepted level in medical physics (Figure 3a for real tissue and b for biomaterial Bragg peak position graphs). It was noted that this difference decreased as the energy increased (with an average of 0.15% at 376 MeV). The secondary peak positions in the hard tissue were found to be similar, and the subsequent decrease in Bragg peak amplitude was approximately similar. At lower energies, the entrance LET values of the Bragg peaks were observed to be higher in real tissue with an average difference of 12.4%. At higher energies, this difference increased up to an average of 14.2%. However, considering the low energy level at which the entrance LET occurs, these differences can be considered negligible. In real tissue, the Bragg peak amplitudes were found to be 15.1% higher at lower energies, on average. As the energy increased, the biomaterials exhibited an increased Bragg amplitude, generating an average difference of 43.2% in LET compared to real tissue. This difference is thought to arise from the atomic and mass density of the biomaterials. Specifically, at low energies, it is normal for heavy ions to create shorter Bragg plateaus within the tissue. However, at higher energies, the extended Bragg plateau and subsequent ionization processes in the denser environment of the hard tissue contribute to this difference.

### 3.2. Recoils

The calculation results in Table 2 indicate that the ^4^He beam underwent atomic-scale interactions as it traversed the layered structure with different densities. High-atomic interactions resulted in the formation of the Bragg peak. The Total Recoils (eV/(Angstrom-Ion)) parameter showed an average difference of 10.3% between the mandible plate phantoms created from tissue and biomaterials. This difference is believed to stem from the atomic diversity given in Table 2. It was observed that high-atomic-number atoms had a lesser impact on recoils due to their lower percentage of occurrence. On the other hand, low-atomic-number atoms, especially hydrogen (H), had a similar and significant impact on recoil interactions due to their higher mass fraction.

### 3.3. Lateral Straggle

**Figure 4:**
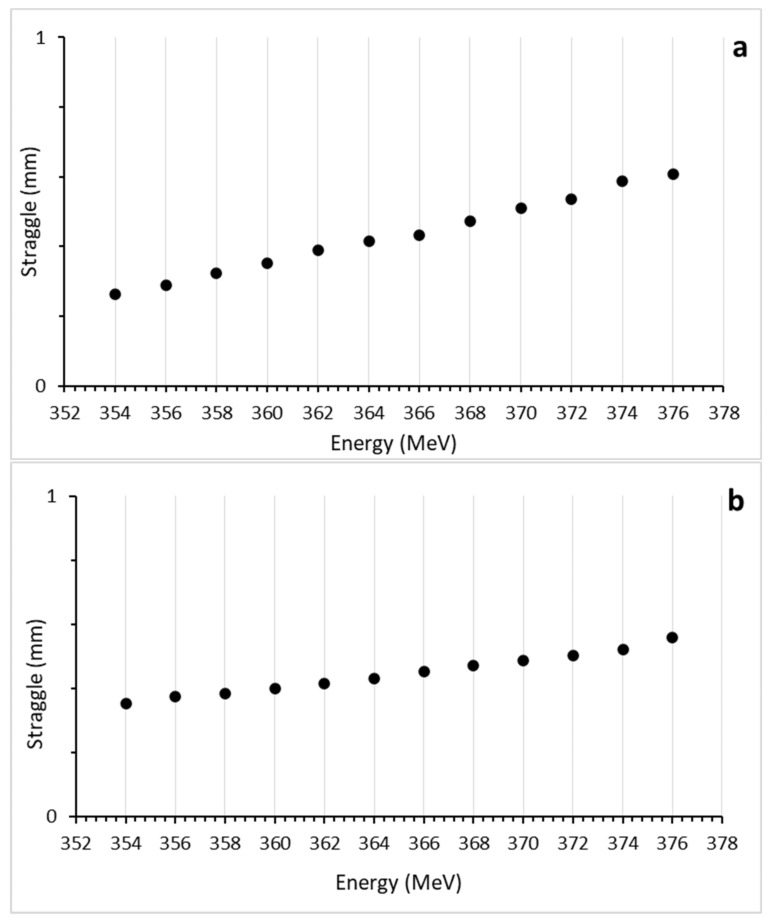
Lateral straggling occurs in the direction of the beam due to interactions between the tissue (**a**) and biomaterial (**b**) mandibular plate phantoms with the 4He ion beam in the range of 354–376 MeV energy.

Figure 4 shows the results of the lateral scattering induced by the ^4^He ion beam in the range of 354–376 MeV of energy for the tissue (a) and biomaterial (b) mandible plate phantoms. The ^4^He ion beam exhibited an average lateral scattering of 0.657 mm with a standard deviation of 0.032 in the tissue mandible plate phantom, showing an increase of 13.7% between the lowest and highest range. In the biomaterial mandible plate phantom, the average lateral scattering was 0.446 mm with a standard deviation of 0.061, and an increase of 36.7% was observed between the lowest and highest range. Considering the average thickness of the cortical and cancellous bone structures (approximately 6 mm), it can be observed that at the energy values (270–276 MeV) that form the Bragg peak positions at the ends of these two structures, a high LET will be delivered to the oral cavity. Therefore, the average lateral scattering should be considered to control lateral scattering within the target.

## 4. Discussion

Helium ions may be a better choice than protons or carbon ions for imaging and treatment monitoring since they have less lateral scattering and require a lower imaging dose [38,39,40]. Among the newly developed systems for reducing range uncertainties for particle beams and for positioning and verification purposes, prompt gamma spectroscopy [41,42,43] and ion beam radiography [44,45,46] can be given as examples. ^4^He ion therapy is still in its early stages, compared to the more mature initial studies. Significant foundational work is needed to develop and investigate ^4^He ions for optimal future applications [5]. Recent studies have taken steps toward evaluating the biophysical events of ^4^He [47], developing RBE (Relative Biological Effectiveness) models [48,49], and evaluating relevant models from a clinical perspective [50]. The RBE prediction may be different for different cases, depending on the endpoints being measured, such as dose, LET, and tissue type [5]. Recent studies are conducting comprehensive dosimetric characterizations for helium ions [11,51] and developing both analytical and MC-based dose engines [39,52]. These studies encompass research and/or clinical investigations, including in vitro studies, treatment plan comparisons, or the development and validation of clinical treatment planning systems. In terms of dose calculation, published studies depict approaches to pencil beam algorithms for ^4^He ions and demonstrate acceptable agreement with both MC simulations and measurements in both homogeneous and heterogeneous environments [39]. This agreement will allow researchers to investigate the parameters that affect the composition, energy, and direction of the radiation field before it hits the target volume. These parameters include electronic energy loss in the patient’s body, multiple Coulomb scattering, and nuclear fragmentation [5]. Additionally, when calculating LET and particle distributions, not only the longitudinal but also the lateral dimension needs to be considered [5,7]. In this regard, helium ions exhibited less lateral scattering in the mandible plate phantom compared to proton beams [14]. To realize the full potential of helium ions, high-performing and validated interaction models and transport codes are needed to accurately define the passage of the helium ion beam through a patient’s body [5,6]. Therefore, the use of MC methods and geometries including representative compositions for materials will help advance the scientific dataset before experimental and therapeutic uses [5,6].

## 5. Conclusions

As a result of clinical, experimental, and simulation studies in the field of heavy ion therapy, a new ion species to be used instead of protons and carbon ions has become the focus of attention. Among these focus areas, helium ions have gained interest. Before their clinical implementation, helium ions should be evaluated in simulation and experimental applications on different phantom types, including various biomaterials, to guide their clinical suitability. Due to the limited availability of such studies, there is still a need for extensive research to understand the benefits and risks of therapeutic helium ion beams. Therefore, it is crucial to use not only water phantoms, but also different biological phantoms composed of tissue or tissue-equivalent materials. Thus, by providing the closest experience to the tissue, the properties of the tissue layers will be brought to the fore. Also, working on a phantom close to real tissue will avoid experimental improvements and reduce the complexity in treatment planning. However, regardless of how much biological material closest to the tissue is used, it is thought that it is difficult to reach the unique structure of the real tissue. Considering the application-oriented nature of helium ion therapy, multidisciplinary collaboration and studies are required to address the discussed issues. This way, it becomes possible to establish the physical, biological, chemical, and clinical foundations of treatment with helium ion beams and uncover the unknowns. To achieve this success, comprehensive and detailed MC-based simulation studies should be conducted due to the limited number of centers equipped with helium ion beams with high energy that are suitable for therapeutic use. In this manner, databases comprising parameters such as range, LET, dose, lateral scattering, and recoils can be established before experimental and treatment measurements, contributing to academic accumulation. The results obtained at the end of this study are listed as follows:

1. Helium ions can be used as intermediate heavy ions in addition to proton and carbon ions.
2. They have more LETs and less lateral scattering than the proton and also cost less than carbon.
3. They have better performance than the proton in the treatment of dental tumors.
4. The biophantom proposed in this study for calibration and dose calculations in dental tumors showed a realistic performance.
5. The biomaterials that make up the biophantom created in this study gave results close to real tissues.

In future endeavors, it is planned to conduct organ-specific ion studies and employ suitable phantom studies for various organs such as the eye, nose, spinal cord, and thyroid.

## Figures and Tables

**Table 1:** Percentage of chemical composition, atomic density, and density of the biological layers that make up the mandible phantom that models the jaw [34].

Layer	Biomaterial	Chemical Composition (%)	Atomic Density (10^22^ atom/cm^3^)	Mass Density (g/cm^3^)
1	Skin	H:10.0, O:59.4, C:25.0, N:4.6, S:0.3, Cl:0.3, P:10.3, Na:0.2, K:0.1	9.88	1.02
	PMMA	H:53.3, C:33.3, O:13.3	8.57	0.95
2	Parotid gland	H:62.5, C:16.4, N:1.27, O:19.6, S: 0.037, Cl:0.016, Na:0.025, P:0.019	10.32	1.02
	PMMA	H:53.3, C:33.3, O:13.3	8.57	0.95
3	SMAS	H:58.3, C:37.4, N:1.45, O:1.89, F:0.532, Ca:0.266	10.65	1.027
	PMMA	H:53.3, C:33.3, O:13.3	8.57	0.95
4	Masseter muscle	H:52.6, C:8.9, N:1.6, O:26.6, S:5.85, Cl:1.76, K:0.64, P:0.404	10.11	1.05
	Paralene_N	H:50, C:50	10.26	1.11
5	Buccal Fat	H:63.4, C:28.4, N:0.304, O:7.77, Cl:0.018, Na:0.011	10.35	0.92
	Polyethylene	H:66.6, C:33.4	12.23	0.95
6	Mucosa	H:10.1, C:77.5, N:3.50, O:5.23, F:1.74, Ca:1.83	5.24	1.028
	PMMA	H:53.3, C:33.3, O:13.3	8.57	0.95
7	Saliva	H:66.6, O:33.3	10.02	1
	Water	H:66.6, O:33.3	10.02	1
8	Gum	H:52.6, C:32.9, N:0.862, O:7.89, Cl:1.72, Mg:3.63	8.88	1
	PMMA	H:53.3, C:33.3, O:13.3	8.57	0.95
9	Cortical bone	H:39.2, C:15.0, N:3.48, O:31.6, S:0.108, P:3.86, Ca:6.53, Mg:9.57	9.94	1.92
	Teflon	C:33.3, F:66.6	7.95	2.2
10	Cancellous bone	H:57.7, C:23.0, N:1.36, O:15.7, S:4.27, P:0.752, Ca:1.26, Fe:1.23	10.42	1.18
	Teflon	C:33.3, F:66.6	7.95	2.2

**Table 2:** The percentage contribution of each atom in the tissue and biomaterial phantoms to the total recoil value (eV/(A-Ion)).

*Phantom*	*Energy*	*Total Recoil*	*Contributions to Recoils of Atoms (%)*												
			H	C	N	O	S	Cl	Na	K	P	Mg	Ca	Fe	F
*Tissue*	354	2.272	23.21	26.13	3.15	26.17	0.13	0.02	0.02	0.02	7.31	3.21	10.67	0.02	
	356	2.521	13.28	15.38	3.21	41.98	0.21	0.02	0.02	0.02	6.62	0.11	19.18	0.02	
	358	2.275	14.71	16.12	3.14	39.52	0.03	0.03	0.03	0.03	8.08	0.17	18.12	0.03	
	360	2.541	15.72	17.12	3.68	39.29	0.24	0.02	0.02	0.02	7.46	0.14	16.28	0.02	
	362	2.332	12.51	19.51	2.78	42.32	0.33	0.05	0.05	0.05	7.15	0.09	15.19	0.05	
	364	2.439	18.41	27.21	2.55	31.46	0.17	0.02	0.02	0.02	6.21	0.08	13.82	0.03	
	366	1.783	31.24	32.48	2.03	28.12	0.14	0.01	0.01	0.01	1.72	0.01	4.22	0.04	
	368	1.730	30.25	34.28	2.64	27.12	0.01	0.01	0.01	0.01	1.54	0.01	4.11	0.04	
	370	1.702	26.23	33.11	6.76	24.39	0.07	0.01	0.01	0.01	5.26	0.01	4.08	0.07	
	372	1.724	30.66	31.08	1.88	29.09	0.14	0.01	0.01	0.01	2.77	0.01	4.31	0.03	
	374	1.890	30.48	32.22	1.68	30.42	0.14	0.01	0.01	0.01	1.62	0.01	3.32	0.13	
	376	0.656	47.71	22.11	1.58	20.72	0.13	0.01	0.01	0.01	1.26	0.01	6.43	0.02	
*Standard deviation*			0.51	9.90	6.90	1.32	6.98	0.08	0.01	0.01	2.61	0.87	5.95	0.03	
*Biomaterial*	354	1.700	29.23	59.19		11.16									0.46
	356	2.516	28.81	50.52		10.24									10.42
	358	1.927	24.82	45.24		17.32									12.62
	360	1.831	20.72	44.62		10.34									24.32
	362	2.995	25.24	42.12		6.86									25.78
	364	1.859	24.22	48.32		6.14									21.32
	366	2.675	22.16	39.46		16.16									22.22
	368	1.829	19.14	46.22		9.12									25.52
	370	2.501	21.42	42.24		14.86									21.52
	372	2.056	18.48	44.72		12.32									24.48
	374	2.725	13.97	52.32		17.42									16.28
	376	1.979	32.52	40.24		7.91									19.32
*Standard deviation*			4.97	5.39		3.81									7.26

## Data Availability

Data sharing not applicable.
